# Competency-Based Reforms in Ayurveda Education: Challenges and Policy Recommendations for the Implementation of the National Commission for Indian System of Medicine (NCISM)

**DOI:** 10.7759/cureus.96653

**Published:** 2025-11-12

**Authors:** Satyajit P Kulkarni, Malhari K Sirdeshpande, Anil A Bhawade, Pallavi S Kulkarni, Girishkumar M Damor

**Affiliations:** 1 Procedural Management (Panchakarma), Manjushree Research Institute of Ayurvedic Science, Gandhinagar, IND; 2 Procedural Management (Panchakarma), Mahatma Gandhi Ayurved College Hospital and Research Centre, Datta Meghe Institute of Higher Education and Research, Wardha, IND; 3 Ayurveda Pharmacology (Dravyaguna), Shree O.H. Nazar Ayurved College, Surat, IND; 4 Clinical Toxicology and Medical Jurisprudence (Agad Tantra Avum Vidhi Vaidyaka), Manjushree Research Institute of Ayurvedic Science, Gandhinagar, IND

**Keywords:** ayurveda, competency-based education, curriculum reform, faculty development, medical education, national commission for indian system of medicine, public health, traditional medicine

## Abstract

Ayurveda, an ancient Indian medical system focused on holistic well-being, faces the challenge of integrating its foundational principles into contemporary healthcare, while adapting to modern scientific paradigms. This paper addresses the critical need for an updated educational framework to preserve traditional knowledge and enhance Ayurveda's integration into the broader healthcare landscape.

This review synthesizes information on recent policy changes, curriculum updates, and pedagogical innovations in Ayurvedic education. It assesses their impact on standardizing and modernizing the training of Ayurvedic practitioners through a qualitative framework, informed by the direct observations and experiences of teaching faculties involved in the National Commission for Indian System of Medicine (NCISM) curriculum implementation.

Significant reforms have established a competency-based curriculum, integrating modern medical sciences, clinical competencies, and research techniques with traditional Ayurvedic texts. Key initiatives encompass practical training modalities, student-centered pedagogical approaches, the provision of elective courses, specialized in-classroom and experiential out-of-classroom training, and the implementation of continuous internal assessment mechanisms.

New regulations also mandate multidisciplinary faculty. However, persistent challenges include inadequate infrastructure, faculty shortages, limited research funding, and state-level variations in educational standards. Disparities in treatment and compensation between government-funded and self-financing institutions further complicate policy implementation.

While Ayurveda education reforms are well-directed, effective implementation faces considerable hurdles. Overcoming these challenges requires collaborative efforts from policymakers, educational institutions, and healthcare stakeholders. The goal is to foster a scientifically rigorous, globally relevant Ayurvedic education system that produces competent professionals capable of contributing to national health objectives and promoting deeper integration of traditional and modern medical practices.

## Editorial

Introduction

The ancient Indian medical system of Ayurveda, rooted in the Vedas, emphasises a holistic approach to health and well-being, integrating mind, body, and spirit to achieve optimal physiological and psychological balance [1,2]. This system, distinct from the disease-centric focus of modern medicine, defines health not merely as the absence of illness, but as a state of equilibrium across an individual's psycho-physiological systems, digestive strength, waste elimination, and immunity [3]. Historically, Ayurveda has been a cornerstone of Indian cultural heritage, and recent efforts have aimed to integrate its principles into mainstream healthcare systems globally [4]. Integration requires an updated, evidence-based educational framework to ensure the preservation of its foundational tenets, while simultaneously adapting to contemporary scientific and medical paradigms. This paper will critically examine recent achievements in reforming Ayurveda education, assessing the extent to which these reforms have fortified its foundational principles and enhanced its integration within the broader healthcare landscape.

While policy changes and curriculum updates in Ayurvedic education have been extensively documented, there is a recognised gap in understanding the ground-level impact and effectiveness of these reforms from the perspective of those directly involved in their day-to-day execution. Therefore, it was necessary to address this by providing a qualitative synthesis of the experiences and insights of teaching faculty amidst the ongoing National Commission for Indian System of Medicine (NCISM) curriculum implementation.

An Overview of Recent Developments, Significant Alterations Implemented Within the Educational Framework

Recent policy changes and curriculum updates have focused on standardizing Ayurvedic education. These efforts aim to integrate modern diagnostic methods and research techniques with traditional Ayurvedic teachings. The establishment of the Ministry of AYUSH by the Government of India in 2014 marked a significant step in promoting and regulating these traditional medicine systems. In 2021, the Indian government established the NCISM, superseding the prior Central Council of Indian Medicine (CCIM). These regulatory bodies have been pivotal in standardizing educational syllabi, promoting research initiatives, and facilitating the incorporation of Ayurveda into the national healthcare framework, thereby cultivating a pluralistic healthcare system. This approach aims to harness the potential of traditional systems like Ayurveda to address unmet healthcare needs and strengthen public health initiatives, as evidenced by its role during health crises [5].

The NCISM is instrumental in this progress, being responsible for upholding educational standards, stimulating research, and supporting the professional growth of Ayurvedic practitioners. To elevate the quality of Ayurvedic education, the NCISM has implemented significant changes, including the development of a new curriculum aligned with international benchmarks, which integrates modern medical sciences, clinical competencies, and research techniques with traditional Ayurvedic texts [6-8]. This integrated curriculum prepares graduates not only to practice Ayurveda effectively but also to engage in interdisciplinary research and collaborate within diverse healthcare settings, thereby bridging the historical divide between traditional and modern medical paradigms. Furthermore, ongoing efforts are directed at developing specialized validation methods tailored to Ayurvedic principles, which are essential for conducting robust randomized controlled trials and advancing interdisciplinary research [9]. This scientific rigor, while respecting the epistemological nuances of Ayurveda, is crucial for gaining broader acceptance and for integrating its profound insights into global health strategies [10].

The updated curriculum emphasizes practical training and clinical exposure, ensuring that future Ayurvedic practitioners possess both theoretical knowledge and hands-on experience, through competency-based medical education approaches [11]. The pedagogical approach has shifted from teacher-centered to student-centered, through the incorporation of non-lecture hours for theoretical and practical learning. This innovative strategy cultivates independent study and improves skill development, via explicit methodologies [12]. The curriculum now mandates dedicated time for self-directed learning, fostering critical thinking and problem-solving skills, essential for navigating complex clinical scenarios [13]. Course and learning outcomes, mapped to program outcomes, have been meticulously framed and implemented across all institutions offering Ayurvedic education, to bring uniformity [14]. Moreover, the integration of modern educational tools and methodologies, such as simulation-based learning and advanced e-learning platforms, further enhances the practical application of Ayurvedic principles in contemporary clinical settings.

A novel idea in this competency-based curriculum is the introduction of elective courses. These online electives provide training in areas like "Ayurveda and Public Health," "Ayurveda and Yoga," and "Ayurveda and Lifestyle Management," thereby broadening the scope of Ayurvedic practice and research relevant to current global health challenges [15,16]. Additionally, specialized training in areas like Agadtantra (toxicology), encompassing the identification of poisons, treatment modalities, and environmental pollution mitigation, reflects a critical expansion of Ayurvedic practice into modern public health concerns. This re-emphasis on fields like Agadtantra highlights the ancient system's potential to address contemporary issues, such as environmental toxicology and public health. The emphasis on the practical application of research within the curriculum, particularly in community medicine and toxicology, signifies a move towards evidence-based medical practice and enhanced public health engagement [17]. Furthermore, the integration of global health perspectives prepares Ayurvedic graduates to contribute to international health initiatives and to address trans-border health disparities, expanding the global reach and relevance of Ayurvedic medicine.

Major changes have been made in the assessment pattern. The curriculum has restructured the evaluation process to incorporate continuous internal assessment and competency-based examinations, moving away from a solely summative-assessment model. This refined framework aims to provide a holistic and accurate evaluation of student learning, promoting consistent engagement with the curriculum and the development of clinical reasoning skills. This ensures the continuous assessment of practical skills, theoretical knowledge, and ethical understanding, preparing students for the multifaceted demands of modern healthcare [18]. The provision for submission of assessment details to a central portal ensures transparency and accountability in the evaluation process, fostering standardized academic rigor across all Ayurvedic institutions. This robust framework is designed to produce highly skilled practitioners and to foster a culture of continuous learning and adaptation within the Ayurvedic community (Table 1).

**Table 1:** Overview of Key Reforms and Anticipated Impacts in Ayurveda Education

Reform	Anticipated Outcome
Reduction in professional years from four to three	This reform has intensified the curriculum, allocating less instructional time to foundational Bachelor of Ayurvedic Medicine and Surgery (BAMS) subjects such as Sharir Rachana, Dravyaguna, Rasashastra Bhaishajyakalpana, and Rognidan-Vikriti Vidnyan, potentially compressing learning for students.
Implementation of integrated teaching methods	The curriculum now supports an integrated pedagogical approach, exemplified by sequential instruction where CNS anatomy is followed by CNS physiology.
Adoption of diverse teaching methodologies	The revised curriculum advocates varied pedagogical strategies beyond lectures, incorporating group discussions, role plays, field visits, and video presentations.
Shift to student-centric focus	Periodic examinations such as MCQs, open-book tests, and small projects promote continuous evaluation.
Inclusion of elective courses	Electives provide online courses in allied subjects, expanding opportunities for interdisciplinary learning.
Enhanced emphasis on research at the undergraduate level	Research is embedded in syllabi, with institutional research committees promoting small-scale projects.

Along with curriculum changes, the NCISM has also made regulatory changes through its new Minimum Standards of Education 2024 [19]. These regulations critically provide for multidisciplinary, qualified faculties (e.g., MSc Anatomy for Assistant Professor in Rachana Sharir, MSc Physiology in Kriya Sharir department, and Master’s in Public Health for Swasthavritta department). This introduces contemporary scientific perspectives into Ayurvedic education, fostering a more integrated and comprehensive understanding of health and disease. This interdisciplinary approach is vital for enhancing the scientific rigor and empirical validation of Ayurvedic principles, aligning them with contemporary biomedical frameworks [20]. Furthermore, the inclusion of faculty with modern medical backgrounds facilitates a richer dialogue between traditional Ayurvedic concepts and current biomedical sciences, ultimately preparing graduates for integrated healthcare settings [21].

Discussion

Challenges and Future Directions for Ayurveda Education Reform

Despite the innovative nature of the revised curriculum, Ayurvedic institutions have encountered significant challenges in their practical implementation at the foundational level. The existing infrastructure, faculty expertise, and opportunities for clinical exposure within many Ayurvedic colleges are frequently inadequate to meet the stringent requirements of the updated curriculum, thereby impeding its effective execution. The shortage of adequately trained faculty, particularly those proficient in both traditional Ayurvedic principles and modern pedagogical techniques, presents a significant barrier to delivering the competency-based education envisioned by NCISM [22]. Furthermore, state-level variations in institutional standards contribute to disparities in educational quality and graduating student competencies nationwide. The focus on government-funded institutions and the differential treatment and compensation scales between government/aided and self-financing institutions complicate the implementation of national policies.

The direct implementation of the curriculum by NCISM, without an initial pilot study, led to widespread confusion and disarray within academic circles. A phased approach, starting with selected institutions before a nationwide rollout, could have yielded valuable insights into potential implementation challenges. Furthermore, despite claims addressing redundancies and repetitions, the new curriculum still contains numerous instances of these issues. It also appears to distance students and faculty from practical realities, potentially having an adverse effect on students' career prospects. For example, the increased emphasis on modern medicine within the curriculum, coupled with unaddressed redundancies, is notable. Specifically, in the Agadtantra curriculum, detailed points regarding post-mortem examinations are included; however, BAMS (Bachelor of Ayurvedic Medicine and Surgery) graduates are legally prohibited from performing autopsies, rendering this theoretical knowledge largely irrelevant for their professional practice [23]. This misalignment between educational content and professional scope necessitates a critical re-evaluation of educational objectives to ensure better synchronization with the actual roles and responsibilities of Ayurvedic practitioners.

The implementation of the new curriculum and the operational dynamics of its establishment committee have been described as exhibiting elements of partiality and centralized control. This approach may, consequently, hinder the cultivation of a collaborative environment, which is crucial for effectively addressing the intricate challenges within Ayurvedic education, and thus could compromise its sustained effectiveness. This centralization also risks stifling innovative pedagogical approaches and curriculum adaptations that could otherwise emerge from diverse institutional contexts.

Concurrently, the NCISM has introduced a new curriculum for postgraduate Ayurveda courses. However, institutions offering these programs frequently lack sufficient, dedicated teaching staff for postgraduate students. This initiative has, consequently, placed a considerable burden on existing faculty and infrastructural resources, thereby compromising the quality of both undergraduate and postgraduate Ayurvedic education [24].

Potential Remedies

NCISM retains the flexibility to continually refine curriculum implementation by actively addressing identified issues and discrepancies. This necessitates a consensual approach to curriculum revisions, incorporating feedback from both faculty and students and engaging institutions across both governmental and private sectors. For instance, while the new Panchakarma curriculum outlines non-lecture-hour methodologies, the NCISM should remain receptive to innovative pedagogical approaches adopted by faculty, provided these demonstrate demonstrable impact and effectiveness. Moreover, a dynamic feedback mechanism should be established to regularly assess the practical applicability and pedagogical efficacy of new curriculum elements, ensuring ongoing refinement and relevance [25].

A more judicious, phased implementation of the new postgraduate curriculum is imperative, coupled with ensuring the sufficient availability of qualified teaching staff. Furthermore, the policy regarding allied teachers in postgraduate education warrants re-evaluation, as its current application may be perceived as both inequitable and inconsistent with academic rigor. Crucially, fostering inclusive participation from all faculties in curriculum development and implementation - free from bias - is essential for elevating the quality of Ayurvedic education to achieve global recognition.

Currently, Rashtriya Ayurved Vidyapeeth (RAV) has been actively involved in the continuing medical education (CME) of Ayurveda teachers and practitioners, providing a vital platform for professional development and knowledge dissemination [26]. If NCISM and RAV collaborate to host the CMEs, focused on the implementation of the new NCISM curriculum, it would significantly enhance the understanding and adoption of the updated educational framework among educators and practitioners alike, addressing critical gaps in knowledge transfer and skill development. This provides a forum for participants to raise concerns about the new curriculum's implementation and propose solutions to the NCISM, which can then address these issues through a collaborative approach.

Conclusion

The policy reforms in Ayurveda education are well-directed and focused; however, their effective implementation faces several challenges. Overcoming these hurdles necessitates a concerted effort from policymakers, educational institutions, and healthcare stakeholders to ensure the effective implementation of these reforms, leading to a more scientifically rigorous and globally relevant Ayurveda education system. Sustained collaboration and curriculum adaptability will ensure Ayurveda’s educational evolution remains both globally credible and contextually grounded. It will ultimately produce a cadre of highly competent Ayurvedic professionals, capable of contributing to national health objectives and fostering a deeper integration of traditional and modern medical practices.
